# MiRNA-203 suppresses cell proliferation, migration and invasion in colorectal cancer via targeting of EIF5A2

**DOI:** 10.1038/srep28301

**Published:** 2016-07-04

**Authors:** Biao Deng, Bin Wang, Jiaqing Fang, Xuchao Zhu, Zhongwei Cao, Qi Lin, Lisheng Zhou, Xing Sun

**Affiliations:** 1 Department of General Surgery, Shanghai General Hospital, Shanghai Jiao Tong University, 100 Haining Road, Shanghai, 200080, China; 2 Department of Gastroenterology, Tianyou Hospital, TongJi University, 500 Zhennan Road, Shanghai, 200331, China; 3 Department of Nuclear Medicine, Shanghai Tenth People’s Hospital, TongJi University, 301 Yanchang Road, Shanghai, 200072, China; 4 Department of Gastroenterology, Shanghai General Hospital, Shanghai Jiao Tong University, 100 Haining Road, Shanghai, 200080, China; 5 Department of General Surgery, Zhongshan Hospital, Fudan University, 180 Xietu Road, Shanghai, 200032, China

## Abstract

While it is known that miR-203 is frequently downregulated in many types of human cancer, little is known regarding its expression and functional role in colorectal cancer (CRC). In this study, we aimed to investigate the expression and the potential mechanisms of miR-203 in colorectal cancer. MiR-203 was significantly downregulated in CRC tissues compared with matched normal adjacent tissues. Our clinical data show that decreased miR-203 was associated with an advanced clinical tumor-node-metastasis stage, lymph node metastasis, and poor survival in CRC patients. Furthermore, externally induced expression of miR-203 significantly inhibited CRC cell proliferation and invasion *in vitro* and *in vivo*. Mechanistically, we identified EIF5A2 as a direct and functional target of miR-203. The levels of miR-203 were inversely correlated with levels of the EIF5A2 in the CRC tissues. Restoration of EIF5A2 in the miR-203-overexpressing CRC cells reversed the suppressive effects of miR-203. Our results demonstrate that miR-203 serves as a tumor suppressor gene and may be useful as a new potential therapeutic target in CRC.

Colorectal cancer (CRC) is ranked as the second most common cancer in women and the third most common cancer in men according to the World Health Organization^1^. As many as 1 million new diagnoses occur every year, resulting in high morbidity and mortality rates worldwide^2^. When diagnosed early with only localized disease, surgery can often be curative. However, only 39.6% of CRC is diagnosed at the localized stage^1^. When detected at advanced stages, after metastasis has occurred, chemotherapy becomes the mainstay of treatment and therapy becomes more complex. Chemoresistance eventually limits the treatment effectiveness, and new targets are needed. Unfortunately, the mechanisms involving CRC tumor aggressiveness and spread are not completely understood.

Recently, there has been a focus on the role of microRNAs in oncogenesis and tumor suppression^3^,^4^,^5^. These small noncoding RNAs are known to regulate protein expression at the post-transcriptional level by binding the 3′-UTR portion of mRNAs to either prevent translation or to promote the degradation of the mRNA^6^. These microRNAs have been described to play a role in a variety of different human cancers, including hepatocellular carcinoma, prostate cancer, cervical cancer, breast cancer and colon cancer, and they are known to play a key role in cell growth and metastasis^7^,^8^,^9^. As such, microRNAs can directly affect tumor growth or tumor suppression.

microRNA 203 (miR-203) was previously reported to act as a tumor suppressor and downregulate many forms of cancer, including hepatocellular carcinoma, leukemia, esophageal cancers, and breast cancer^10^,^11^,^12^,^13^,^14^. More recently, it has been evaluated in prostate cancer and is noted to be an important determinant of tumor proliferation and invasion^15^,^16^. However, some studies showed that the upregulation of miR-203 expression was found in ovarian cancer^17^. In CRC, some studies showed an increase in miR-203 expression^18^, while other studies showed a decrease in miR-203 expression^19^,^20^,^21^. Previous reports suggest that miR-203 plays a role in chemoresistance via downregulation of Akt2 gene expression, which is implicated in the expression of the MTDH and HSP90 genes^22^. Clear evidence indicates that miR-203 acts as tumor suppressor in CRC. However, the molecular mechanisms of miR-203 in colorectal cancer remain largely unclear.

Eukaryotic initiation factor 5A2 (EIF5A2), located on chromosome 3q26, has also been described to be involved in a variety of cancers, including colorectal cancer, hepatocellular cancer, ovarian cancer, and bladder cancer^23^. There are reports depicting the expression and oncogenic role of EIF5A2 in hepatocellular carcinoma, and EIF5A2 has been suggested to modulate several pathophysiological processes by promoting cell proliferation and cell metastasis by inducing epithelial-mesenchymal transition (EMT)^24^,^25^. Zhu *et al*. studied clinical samples from CRC patients and found that EIF5A2 might be involved in CRC aggressiveness and that EIF5A2 increased the expression of MTA1 to induce EMT^26^.

In this study, we found that reduced expression of miR-203 in CRC is important in the acquisition of an aggressive and poor prognostic phenotype. The re-expression of miR-203 significantly suppresses cell motility and invasiveness in *in vitro* and *in vivo* assays. The molecular mechanism of miR-203 involving in functional target was also addressed in this study.

## Results

### MiR-203 is downregulated in CRC tissues

**Figure 1:**
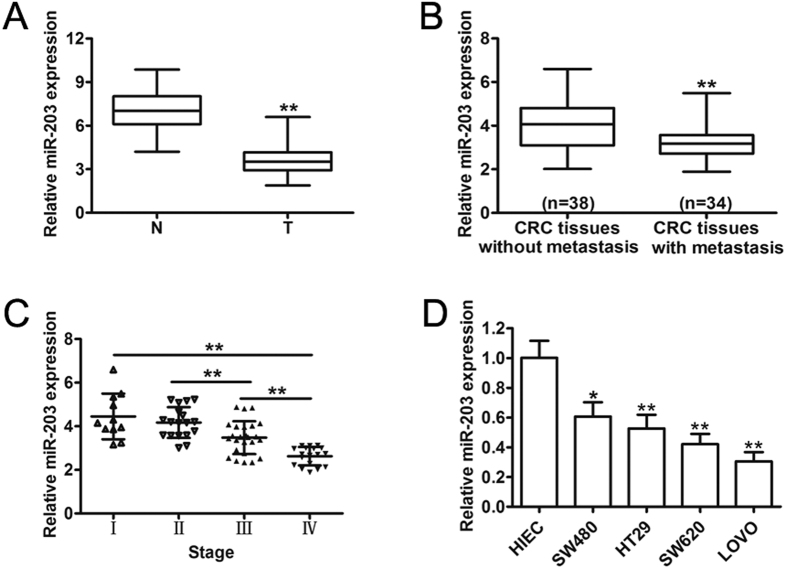
miR-203 was downregulated in the CRC clinical specimens and the human CRC cell lines. (**A**) miR-203 expression was determined by qRT-PCR in 72 pairs of CRC tissue (T) and the corresponding adjacent non-tumor tissue (N). (**B**) The miR-203 expression level was investigated in primary CRC tissues with metastasis or without metastasis. (**C**) Assessment of miR-203 levels from the total RNA derived from CRC tissues according to the tumor stage. (**D**) The relative expression of miR-203 in the CRC cell lines compared with the normal human intestinal epithelial cell line HIEC. *p < 0.05, **p < 0.01.

To further investigate the expression of miR-203 in CRC, 72 pairs of CRC tumor tissues and their corresponding adjacent non-tumor tissues were compared by qRT-PCR. The results showed that miR-203 expression was significantly lower in CRC tumor tissues compared to adjacent non-tumor tissues (Fig. 1A). A reduction in the levels of miR-203 in the primary tumors with metastasis was also noted compared to the primary tumors without metastasis (P < 0.05) (Fig. 1B), which suggested that the downregulation of miR-203 was closely related to the increase of CRC metastasis. Furthermore, to identify whether miR-203 is perturbed, not only during cancer initiation but also during CRC progression, we examined the expression levels of miR-203 at different stages of CRC. We found that miR-203 levels decreased during CRC progression (Fig. 1C). We further examined miR-203 expression in a series of human CRC cell lines and a normal HIEC line. Consistent with the data obtained from the CRC clinical specimens, miR-203 was downregulated in all of the CRC cell lines compared with the normal HIEC line (Fig. 1D). Interestingly, the SW620 and LOVO cells, which have high metastatic capacities, expressed the lowest levels of miR-203. These results suggested that miR-203 is decreased in CRC and might play an important role in CRC progression.

### Lower levels of miR-203 are associated with poor clinicopathological features and prognosis in CRC

**Figure 2:**
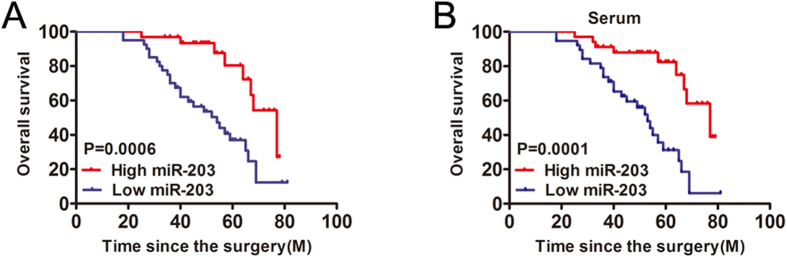
Survival curves of the CRC patients according to miR-203 expression. (**A**) Kaplan-Meier curves for the overall survival curves of the patients with CRC expressing low and high amounts of miR-203. (**B**) Kaplan-Meier curves for the overall survival curves of the patients with CRC according to the expression of miR-203 in the serum. The median value was used to distinguish high expression and low expression.

To evaluate the clinical significance of miR-203 expression, we divided the CRC patients into two groups (low and high miR-203 expression) with the median miR-203 expression levels serving as the cutoff point between the two groups. The statistical analysis of the 72 cases of CRC revealed that lower levels of miR-203 were significantly associated with the clinical tumor-node-metastasis stage and lymph node metastases (P < 0.01) (Table 1). There was no statistically significant association between miR-203 expression and the other clinicopathological parameters, including age, gender, tumor size, location, and histological grade. To understand the prognostic significance of miR-203 in CRC, a Kaplan-Meier survival analysis was performed to assess the relationship between the expression of miR-203 and the overall survival. The results showed that reduced miR-203 levels were significantly associated with shorter overall survival times (P < 0.001) (Fig. 2A). The median survival time was 54 months in the miR-203 low group (n = 40) and 77 months in the miR-203 high group (n = 32). Interestingly, the patients with higher plasma miR-203 levels had a better overall survival (Fig. 2B). Moreover, a univariate analysis showed that miR-203, lymph node metastasis, and tumor-node-metastasis stage were significantly associated with overall survival (OS) in CRC patients (Table 2). A multivariate analysis showed that miR-203 was an independent prognostic indicator for OS (Table 2).

### MiR-203 suppresses CRC tumor growth *in vitro* and *in vivo*

**Figure 3:**
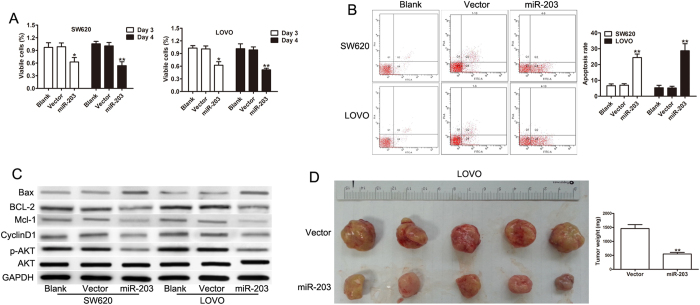
miR-203 inhibited cell growth and tumor growth. (**A**) The proliferation rate of the miR-203 and the vector transfected cell lines as determined by the Cell Proliferation Assay Kit. (**B**) Cell apoptosis in SW620 and LOVO cells by flow cytometric assay. (**C**) The expression levels of Mcl-1, Bcl-2, cyclin D1, Bax, p-AKT and AKT were detected in the SW620 and LOVO cells transfected with the control or miR-203. (**D**) Representative images of the LOVO cell tumors expressing miR-203 or the vector. A quantitative analysis of the weight of the subcutaneous tumors from the two groups. *p < 0.05, **p < 0.01.

To determine whether miR-203 had an effect on the malignant phenotype of CRC cells, we established SW620 and LOVO colon cancer cell lines that stably express miR-203. As shown in Supplementary Figure 1, the level of miR-203 was markedly increased in the stable cell lines compared with the control cells. Next, a series of biological experiments were performed to test the effect of miR-203 on cell growth, including proliferation and apoptosis assays. The overexpression of miR-203 significantly inhibited the proliferation of CRC cells (Fig. 3A). The apoptosis assays demonstrated that miR-203 overexpression significantly promoted apoptosis in SW620 and LOVO cells (Fig. 3B). The inhibition of growth and the induction of apoptosis was further confirmed by the expression of proliferation and apoptosis related genes using a western blot. As shown in Fig. 2C, the protein levels of Bcl-2, Mcl-1, cyclin D1, and p-AKT were decreased, but Bax was increased in SW620 and LOVO cells that stably overexpress miR-203 (Fig. 3C). Consistent with the *in vitro* results, the overexpression of miR-203 in LOVO cells significantly suppressed the overall tumor growth *in vivo* (Fig. 3D). Conversely, the effective knockdown of miR-203 with miR-203 inhibitors significantly enhanced the proliferation (Supplementary Figure 2A,B) and decreased the apoptotic levels (Supplementary Figure 2C) of SW480 cells, which have a relatively higher endogenous miR-203 expression. These data indicate that miR-203 might act as a tumor suppressor and that its over-expression in cancer cells might inhibit cell proliferation.

### MiR-203 inhibits CRC migration and invasion *in vitro* and *in vivo*

**Figure 4:**
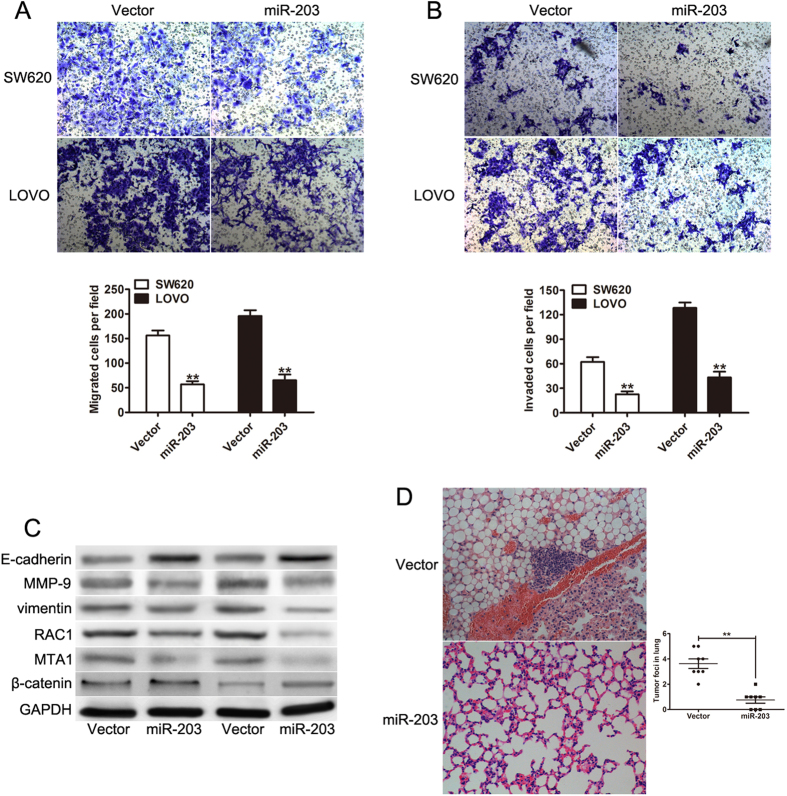
miR-203 suppresses cell migration and invasion *in vitro* and *in vivo*. (**A**) Representative pictures show the migrated SW620 and LOVO cells (left) and quantification of the number of migrated tumor cells (right). (**B**) Representative pictures of the matrigel invasion assay were obtained for the SW620 and LOVO cells (left), and the number of invasive tumor cells was quantified (right). (**C**) Western blot analysis of the relative protein levels of MTA1, RAC1, MMP-9, vimentin, β-catenin and E-cadherin in the miR-203 and control groups for both the SW620 and LOVO cell lines. (**D**) Representative hematoxylin and eosin staining of the lungs isolated from mice injected with the LOVO-vector or LOVO-miR-203 cells. The number of metastatic nodules in the lung was counted in 8 randomly selected high-power fields under a microscope. **p < 0.01.

Given that the expression of miR-203 is associated with the metastatic property of CRC, we wondered whether miR-203 plays a potential role in CRC cell invasion and metastasis. Transwell assays without Matrigel suggested that miR-203 dramatically inhibits the migration of SW620 and LOVO cells when compared with the vector groups (Fig. 4A). Transwell assays with Matrigel also showed that the invasive capacities were significantly decreased in these two stable cell lines when compared with the vector cells (Fig. 4B). We then examined the alteration of metastasis related genes that were crucial to the cell malignant phenotypes. Our results indicated that miR-203 induced the expression of E-cadherin and β-catenin but repressed the expression of MMP-9, vimentin, MTA1 and RAC1 (Fig. 4C). To further explore the role of miR-203 in tumor invasion and metastasis *in vivo*, we injected LOVO-miR-203 cells that were stably expressing miR-203 or vector-transfected control cells into nude mice through the tail vein. The number and size of the metastatic nodules in the lungs was dramatically decreased in the miR-203 groups when compared with the vector controls (Fig. 4D). In contrast, the migration and invasiveness of SW480 cells increased when endogenous miR-203 was silenced with a miR-203 inhibitor (Supplementary Figure 3). Taken together, these observations indicated that miR-203 significantly inhibits CRC cell migration and invasion *in vitro* and *in vivo*.

### MiR-203 directly targets EIF5A2 in CRC cells

**Figure 5:**
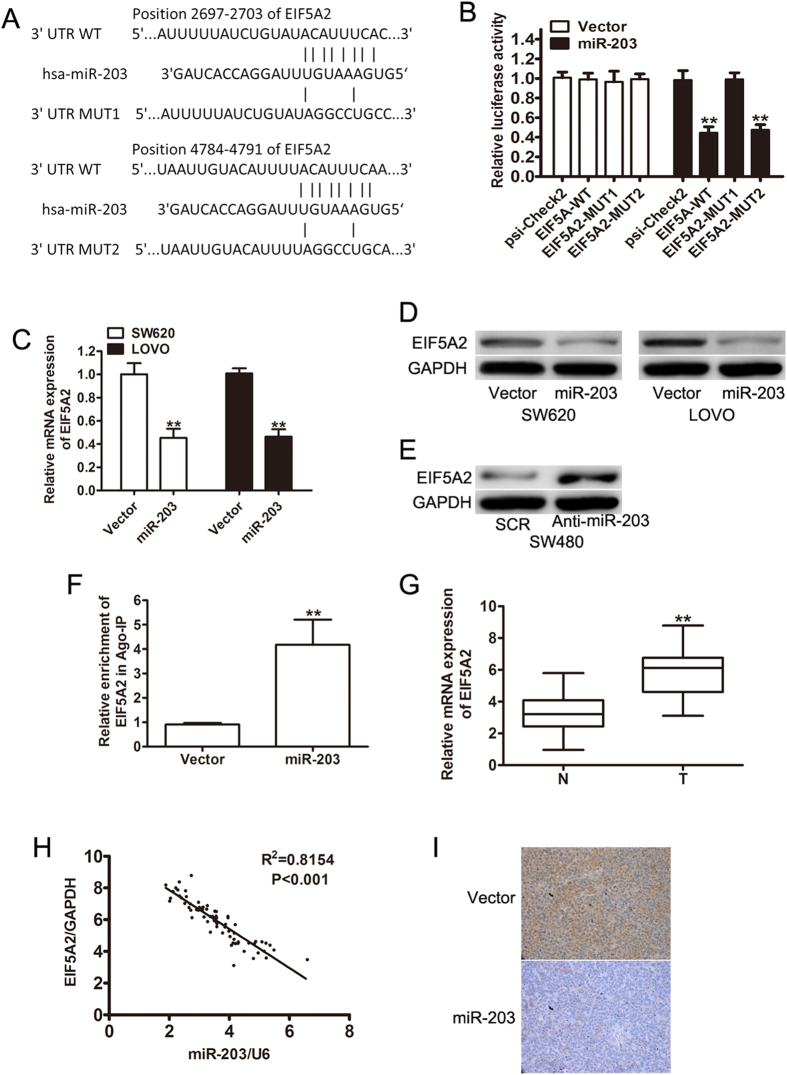
miR-203 directly targets EIF5A2. (**A**) The putative miR-203-binding sequence in the 3′UTR of the EIF5A2 mRNA is shown. A mutation was generated in the EIF5A2 3′UTR sequence at the complementary site for the seed region of miR-203. (**B**) The luciferase reporter assays show reporter activity after co-transfection of either EIF5A2-3′-UTR or mutant 3′UTRs of EIF5A2 with miR-203 in the HEK-293 cells (**P < 0.01). (**C,D**) The re-expression of miR-203 in the SW620 and LOVO cells attenuated the expression of EIF5A2 mRNA and protein levels, respectively. (**E**) A western blot analysis of the relative protein levels of EIF5A2 in the miR-203 inhibitor and control inhibitor groups in the SW480 cell lines. (**F**) RIP-IP assays were performed to co-IP the Ago2 complexes from the LOVO cells transfected with the control or miR-203. The relative enrichment of the EIF5A2 mRNA from the Ago2 co-IP fractions was detected by qRT-PCR assays. (**G**) The relative level of EIF5A2 mRNA in 72 pairs of CRC and adjacent nontumor mucosa tissues. (**H**) A Spearman Correlation analysis clearly shows a negative correlation between miR-203 and EIF5A2 mRNA expression in the CRC tissues (P < 0.001). (**I**) The IHC staining shows EIF5A2 in the tumor samples from the LOVO-vector and LOVO-miR-203 cells. **p < 0.01.

To elaborate the molecular mechanisms by which miR-203 suppresses CRC growth and metastasis, we searched for candidate target genes of miR-203 that might be involved in the pathogenesis of CRC. The bioinformatic algorithm TargetScan was used to predict EIF5A2 as a putative target for miR-203, which might contribute to its tumorigenic functions in CRC (Fig. 5A). To verify the in silico prediction, luciferase reporter constructs carrying the 3′ UTR miR-203 potential-binding site or one of two mutant-binding sites of EIF5A2 were created and co-transfected with miR-203 or a vector into HEK293T cells. The luciferase assays revealed that the overexpression of miR-203 significantly reduced the luciferase activity of the EIF5A2-WT (P < 0.01) and EIF5A2-MUT2 (P < 0.01) luciferase reporters when compared with the vector (Fig. 5B). miR-203 did not have an effect on the luciferase activity of the EIF5A2-MUT2 luciferase reporter (Fig. 5B), which indicated that miR-203 mediated the expression of EIF5A2 by binding the 2697–2703 nucleotide of the EIF5A2 3′ UTR. Moreover, the overexpression of miR-203 led to a significant decrease in EIF5A2 expression at both the mRNA (Fig. 5C) and protein levels (Fig. 5D). In contrast, the downregulation of miR-203 with a miR-203 inhibitor resulted in a significant increase in the expression of EIF5A2 in SW480 cells (Fig. 5E). To further confirm that miR-203 directly interacts with EIF5A2, an RNA pull-down assay was utilized to explore the miR-203-mediated binding of RISC to the EIF5A2 mRNA. As shown in Fig. 5F, the Ago2 co-IP fractions from the LOVO-miR-203 cells were significantly enriched for the EIF5A2 mRNA compared to the vector-transfected control cells. We then used qRT-PCR to detect the expression level of EIF5A2 in CRC tissues and adjacent non-tumor mucosal tissues. Compared with the adjacent non-tumor mucosal tissues, the EIF5A2 mRNA was significantly upregulated in 72 CRC tissues and this upregulation was strongly correlated with the downregulation of miR-203 (Fig. 5G,H). Additionally, the protein levels of EIF5A2 in tumors tissues from mice with a subcutaneous LOVO implantation were analyzed using immunohistochemical staining. Low EIF5A2 staining was observed in the CRC tissues from the subcutaneous implantation models of LOVO stably transfected with miR-203 compared with the controls (Fig. 5I). These findings indicated that miR-203 might negatively regulate the expression of EIF5A2 by directly targeting its 3′-UTR. In conclusion, our data further confirmed that miR-203 suppressed tumor growth and invasion by repressing EIF5A2 expression.

### EIF5A2 is involved in miR-203-mediated CRC cell proliferation, migration and invasion

**Figure 6:**
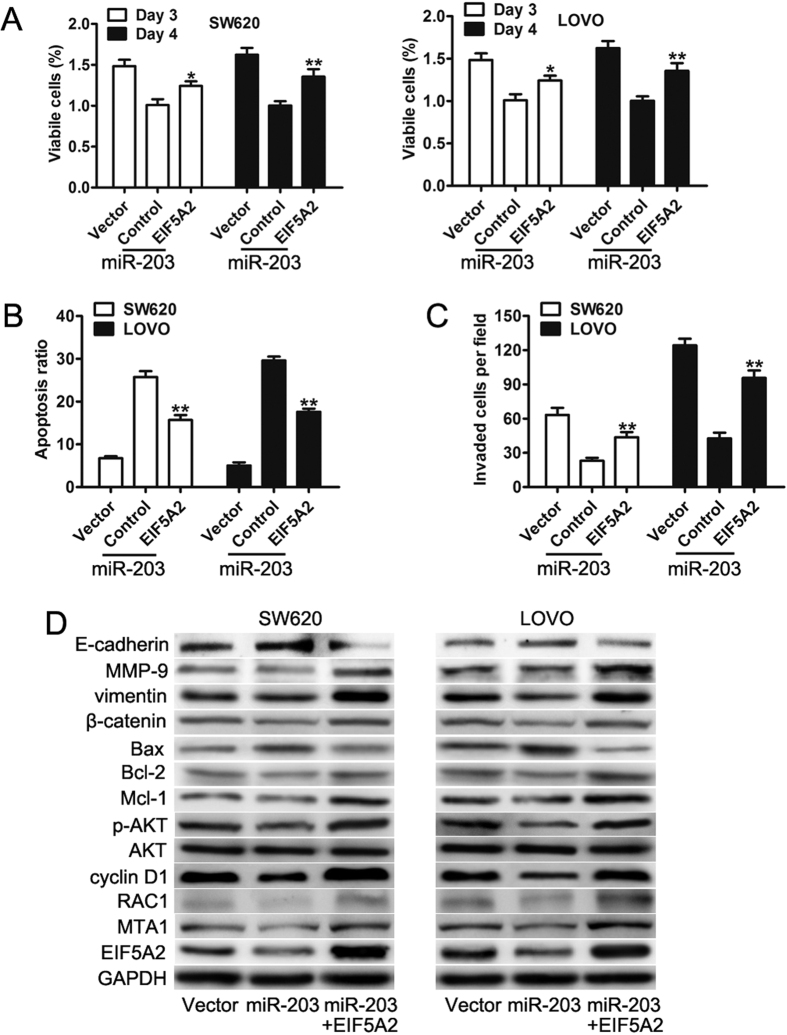
The effects of miR-203 overexpression can be reversed by the induction of EIF5A2. (**A**) Proliferation analysis of cell growth rates as indicated. (**B**) Quantification of the migrated cells as determined by the migration assay. (**C**) Quantification of the invasive tumor cells as determined by the matrigel invasion assay. (**D**) Western blot to detect EIF5A2, MTA1, RAC1, Mcl-1, Bcl-2, cyclin D1, Bax, p-AKT, AKT, MMP-9, vimentin, β-catenin, E-cadherin, and the loading control GAPDH in the SW620, LOVO, and SW480 cells as indicated. *p < 0.05, **p < 0.01.

Previous reports have shown that EIF5A2 is frequently upregulated in CRC and promotes CRC cell migration and invasion^26^, and our results suggest that miR-203 downregulates the expression of EIF5A2 at the mRNA and protein levels by directly binding to its 3′ UTR. Therefore, we hypothesized that the up-regulation of EIF5A2 directly mediates miR-203-medicated cancer biology. To further elaborate on this critical issue, we forced EIF5A2 expression in SW620 and LOVO cells stably expressing miR-203, which was confirmed by a western blot analysis (Fig. 6D). The ectopic EIF5A2 expression in the miR-203-transduced cells attenuated the inhibitory effect of miR-203 on CRC growth (Fig. 6A,B). Similarly, the restoration of EIF5A2 significantly promoted HCC cell invasion, which was inhibited by miR-203 (Fig. 6C). In addition, the attenuated expression of Bcl-2, Mcl-1, cyclin D1, p-AKT, MMP-9, vimentin, MTA1 and RAC1 and the upregulated expression of E-cadherin, Bax and β-catenin was reversed by the overexpression of EIF5A2 (Fig. 6D). Taken together, these results indicated that EIF5A2 was a direct functional downstream target of miR-203 in CRC cells.

## Discussion

There is increasing evidence that dysregulation of miRNAs is involved in colorectal cancer. MiR-203 has been shown to regulate the biological function of cancer cells, including cell proliferation, migration, invasion and chemoresistance, by targeting several target genes, such as CKAP2, LASP1, BIRC5, WASF1, ASAP1, SNAI2, and RUNX2 in prostate cancer^15^,^27^ and AKT2 in CRC^22^. Whether miR-203 is involved in the malignant behavior of human colorectal cancer cells remains unclear. In this study, we revealed that miR-203 expression was significantly lower in CRC tissues and was associated with tumor-node-metastasis stage, lymph node metastasis and a poor prognosis in CRC patients. Moreover, we also found that the overexpression of miR-203 significantly suppressed tumor growth, invasion and metastasis of CRC by targeting EIF5A2 *in vitro* and *in vivo* assays. This study is the first to investigate the role of miR-203 in the proliferation and metastasis of CRC cells *in vitro* and *in vivo*.

In this study, we first confirmed that the expression of miR-203 in CRC tissues was significantly lower than in adjacent non-tumor mucosa tissues. The downregulation of miR-203 was correlated with an increased and more-advanced TNM stage and lymph node metastasis. In addition, CRC patients with low miR-203 expression had worse prognoses than did patients with a high miR-203 expression. This result is consistent with some reports showing a down-regulation of miR-203 in the development of cancers^10^,^13^,^16^,^19^. However, other reports found that miR-203 was overexpressed and acted as an oncogene in bladder cancers^28^ and pancreatic adenocarcinoma^29^. The controversial results suggested that the functions of miR-203 were possibly related to its tissue- and time-dependent expression during distinct cellular processes and the expression levels of its target genes. Further experiments revealed that miR-203 is anti-proliferative and anti-invasive and its proapoptotic effects occur through alterations of the PI3K/Akt and β-catenin pathways and multiple genes.

To further explore the biological functions of miR-203 in CRC cells, we determined the effect of miR-203 on cell growth and invasion. Further studies demonstrated that the overexpression of miR-203 suppresses *in vitro* cell proliferation and invasion, promotes apoptosis, and inhibits *in vivo* tumor growth and metastasis. These results are consistent with findings in prostate cancer and gastric cancer, in which miR-203 was down-regulated, and the ectopic expression of miR-203 suppressed cell proliferation and invasion^15^,^30^,^31^. In addition, the overexpression of miR-203 enhanced the sensitivity of paclitaxel to p53-mutated colon cancer cells. These results demonstrated that miR-203 inhibited not only tumor growth and metastasis but also reversed chemoresistance in CRC. Recent studies have shown that miRNAs play a key role in host-virus interactions during the progression of cancers^32^,^33^,^34^, but the role of miR-203 in viral/host gene regulation and its underlying molecular mechanism needs to be further explored.

We further identified EIF5A2 as a downstream target gene of miR-203 using a luciferase assay and a western blot. EIF5A2 has been implicated as a key contributor to cell motility and tumor metastasis in diverse cancers^23^. Zhu *et al*. reported that EIF5A2 was upregulated in colorectal carcinoma tissues and the overexpression of EIF5A2 enhanced CRC cell motility and invasion by enhancing the enrichment of c-myc on the promoter of MTA1^26^. Tang *et al*. have also found an apparently higher expression of EIF5A2 in hepatocellular carcinoma, and EIF5A2 promoted HCC cell motility and invasion *in vitro* and *in vivo* by activating RhoA/Rac1 to stimulate the formation of stress fibers^24^. An EIF5A2 increase has also been reported in esophageal squamous cell carcinoma and was shown to promote cell migratory and invasive abilities via the HIF1α-mediated signaling pathway^35^. Consistent with previous results, we found that EIF5A2 mRNA expression was significantly higher in the CRC tissues than that in the non-tumor mucosa tissues. A significant inverse correlation between the levels of miR-203 and EIF5A2 was also observed in the CRC tissues by a Pearson correlation analysis. In addition, the overexpression of EIF5A2 markedly abrogated miR-203-mediated suppression of these events, indicating that miR-203 regulates CRC proliferation and invasion, at least partly, by directly blocking EIF5A2 expression.

In summary, our findings demonstrated that miR-203 is down-regulated and is inversely related to TNM stage and lymph node metastasis in CRC. The overexpression of miR-203 inhibited CRC growth and invasion via a repression of EIF5A2 *in vitro* and *in vivo*. These findings indicate that miR-203 is a tumor suppressor gene and represents a potential therapeutic target and prognostic marker for CRC.

## Methods

### Patients and clinical specimens

A total of 72-paired CRC primary tumors and their corresponding non-tumor mucosa were collected between September 2005 and May 2008 following surgical resection at the Shanghai First People’s Hospital of Shanghai Jiao Tong University. Written informed consent was obtained from all patients involved, and the CRC diagnosis was based on hematoxylin and eosin (H&E) stained tumor tissue sections. All of the samples were obtained from patients who had received no preoperative adjuvant chemotherapy or radiotherapy. The demographic and disease data were collected from each patient, including age, gender, tumor size, histologic grade, TNM stage at diagnosis, location, lymph node status, the presence of metastatic disease, and survival. This study was carried out in accordance with the Helsinki declaration and approved by the Medical Ethics Committee of the Shanghai General Hospital of Shanghai Jiao Tong University with the permit number of SHJT-121614X.

### Cell culture

The normal human intestinal epithelial cell line (HIEC) and the human embryonic kidney cell line 293 T (HEK293T) were conserved in our own laboratory. Four human CRC cell lines (SW480, HT29, SW620 and LOVO) were purchased from American Type Culture Collection. All of the cell lines were maintained in RPMI 1640 medium (Invitrogen, Carlsbad, CA, USA) or Dulbecco’s Modified Eagle Medium (DMEM, Invitrogen) supplemented with 10% fetal bovine serum (FBS, Invitrogen) at 37 °C in a 5% CO_2_ incubator.

### RNA extraction and quantitative real-time PCR (qRT-PCR)

Total RNA from the frozen tissues, 1 ml of serum and the cultured cells was extracted using the TRIzol reagent (Life Technologies Corporation, Carlsbad, CA, USA) according to the manufacturer’s instructions. RNA was quantified using a NanoDrop 2000 (Nano-drop Technologies, Wilmington, DE, USA) and stored at −80 C. For miR-203 expression, a stem-loop reverse transcription-polymerase chain reaction (RT-PCR) was carried out using an All-in-One™ miRNA quantitative RT-PCR (qRT-PCR) Detection Kit (GeneCopoeia, Rockville, MD, USA) according to the manufacturer’s protocols. Real-time PCR was carried out with SYBR green detection with a forward primer for the mature miRNA sequence and a universal adaptor reverse primer. U6 small nuclear RNA was used as an internal control.

To detect the EIF5A2 mRNA expression, first-strand cDNA was first synthesized from 500 ng of RNA using the Prime-Script RT reagent kit (TaKaRa, Dalian, China) followed by PCR amplification in an ABI 7900HT real-time PCR system (Life Technologies) as directed by the manufacturer’s protocol. The EIF5A2 gene was amplified by the forward primer: 5′-GGACGACCATGCAAAATAGTGG-3′ and the reverse primer: 5′-TGCCCGTGAAAATATCAATTCCA-3′. The GAPDH gene was amplified by the forward primer: 5′-AGCCTTCTCCATGGTGGTGAA-3′ and the reverse primer: 5′-ATCACCATCTTCCAGGAGCGA-3′. GAPDH was used as an internal control. Each measurement was carried out in quadruplicate, and the experiments were repeated at least three times.

### Construction of vectors and cellular transfection

The sequence containing the pre-miR-203 was amplified by PCR and inserted into the lentiviral plasmid pCDH-CMV-EF1-copGFP vector (System Biosciences, Mountain View, CA, USA) to generate pCDH-miR-203 (miR-203). An empty vector was used as a control (vector). The EIF5A2 cDNA was amplified by PCR and cloned into the pcDNA3.1 (+) (Life Technologies). An empty control vector was used as a control (Control). All of the constructs were verified by sequencing.

The lentiviral constructs expressing miR-203 were packaged with the pPACKH1 lentivector packaging kit (SBI) in HEK293T cells using the X-tremeGENE HP DNA Transfection Reagent (Roche, Basel, Switzerland). The virus particles were harvested 48 h after transfection. SW620 and LOVO cells were infected with recombinant lentivirus-transducing units supplemented with 8 mg/ml Polybrene (Sigma, St Louis, Missouri, USA).

pcDNA3.1-EIF5A2 or the control plasmid pcDNA3.1(+) and inhibitors of miR-203 and scrambled oligonucleotide controls were transfected into CRC cells in 6-well plates using the X-tremeGENE HP DNA Transfection Reagent according to the manufacturer’s instructions.

MiRNA negative control (scramble control inhibitor) and anti-miR-203 were obtained from Dharmacon (Austin, TX, USA) and transfected with the X-tremeGENE HP DNA Transfection Reagent (Roche) in SW480 cells at a final concentration of 50 nM. qRT-PCR analyses were used to confirm the knockdown efficacy of miR-203 after a 48 h transfection.

### Bioinformatics and luciferase reporter assay

The software programs TargetScan (http://www.targetscan.org/mamm_31/) and miRanda (http://www.microrna.org/microrna/home.do) were used to predict the potential target genes of miR-203. The nucleotide sequence of the 3′ untranslated region (UTR) of EIF5A2 was identified as a potential target gene, and a luciferase reporter construct was created to assess and verify the transcriptional activity in the transfected cells.

To create a 3′-UTR luciferase reporter construct of EIF5A2, a wild-type 3′UTR segment of EIF5A2 that contains putative miR-203 binding sites was amplified and cloned into the XhoI and NotI sites downstream of the luciferase reporter gene in psiCHECK-2 (Promega, Madison, WI, USA). The following primers were used to amplify the 3′-UTR of EIF5A2: forward primer: 5′-CCCCCTCGAGACGGAAACATCAGGCATGAAC-3′, reverse primer: 5′-AAAAGCGGCCGCCAACTGATGAAGTTAACAGACAAAT-3′. We conducted mutagenesis of the 3′-UTR in the miR-203 seed complementary site as described previously^36^. Wild-type and mutant luciferase reporter plasmids were confirmed by sequencing. The luciferase activity was measured 48 hours after the transfection using a Dual-luciferase assay system (Promega) according to the manufacturer’s instructions. The renilla luciferase activity was normalized to the Firefly luciferase activity.

### RNA Binding Protein Immunoprecipitation (RIP) assay

The RIP assay was performed using the RIP™ RNA-Binding Protein Immunoprecipitation Kit (Millipore, Billerica, MA, USA) following the manufacturer’s instructions. The Ago2 antibody (Millipore) was used for the RIP, and the SNRNP70 antibody (Millipore) was used as positive control for the RIP procedure. The purified RNA was subjected to a qRT-PCR analysis to assess the presence of the binding targets using special primers as follows: forward primer: 5′-CCATGTATAGAATGTGGACTG-3′, reverse primer: 5′-CTATGTTGTAGCAAATAAGGTAT-3′.

### Cell proliferation assay

The cell proliferation rate was measured using the Cell Titre^®^ 96 Aqueous One Solution Cell Proliferation Assay Kit (Promega, Madison, WI, USA). The cells were seeded at a density of 3000 cells/well in a 96-well plate, 20 μl of MTS (Promega) was added into the medium at different time points and the absorbance (450 nm) was assessed on an ELISA plate reader (Tecan Group Ltd, Männedorf, Switzerland).

### Apoptosis assay

The cells were harvested, washed, and stained with annexin V-PE and propidium iodide using the Annexin V-PE Apoptosis Detection Kit I (BD, Franklin Lakes, NJ, USA) according to the manufacturer’s protocol. Then, the stained cells were measured by flow cytometry (BD FACSCalibur) and analyzed using Kaluza software version 1.2.

### Migration and invasion assays

The transwell migration and invasion assays were performed using standardized protocols as described earlier^37^. Briefly, 3 × 10^4^ cells in 100 μl of serum-free media were added to the upper chamber of an insert (8-μm pore size, millepore) coated with or without Matrigel (BD). Six hundred μl of media containing 10% FBS were added to the lower chamber. The cells were fixed and stained with 0.05% crystal violet after 12 hours or 36 hours for the migration and invasion assays, respectively. Six random fields of each chamber were photographed using an IX71 inverted microscope (Olympus, Tokyo, Japan) at 200x magnification.

### Western blot analysis of protein

Total protein was extracted from the cells using RIPA lysis buffer (Cell Signaling Technology, Beverley, MA, USA). Equal amounts of protein were separated by a 10% SDS-PAGE and transferred to nitrocellulose membranes (Millipore, Billerica, MA, USA). The membranes were blocked with 5% milk in TBST for 2 h at room temperature and probed with the appropriate primary antibody overnight at 4 °C and then with the appropriate horseradish peroxidase-conjugated secondary antibody. The following antibodies were used: EIF5A2, Mcl-1 (Epitomics, Burlingame, CA, USA), BCL-2 (Proteintech, Chicago, IL, USA), E-cadherin, MMP-9, MTA1, CyclinD1 (Abcam, Cambridge, MA, USA), RAC1 (Millipore, Billerica, MA, USA), Phospho-Akt (Ser473), AKT, β-catenin, Bax, Vimentin, and GAPDH (CST, Beverly, MA, USA). The detection of GAPDH on the same membrane was used as a loading control.

### *In vivo* studies

The animal experiments were approved by the Institutional Review Board of the Shanghai General Hospital of Shanghai Jiao Tong University with the permit number of SHJT-1406DB and were undertaken in accordance with the National Institutes of Health Guide for the Care and Use of Laboratory Animals. We used four-week-old BALB/c nude mice for the CRC xenograft models. Medium (150 ul) containing 1 × 10^7^ CRC cells was injected subcutaneously into the left posterior flank regions of each mouse. The mice were sacrificed after 28 days, with subsequent dissections, excisions, and measurements of the mass of the primary tumors.

Six-week-old BALB/C-nu/nu nude mice were purchased from the Shanghai Laboratory Animal Center of China. For the tumor metastasis assays, LOVO cells transduced with lentiviral constructs carrying either miR-203 or the vector control were harvested and washed and were re-suspended in 0.2 ml serum-free RPMI1640 with 2 × 10^6^ cells. The suspended cells were injected into the lateral tail vein of each mouse. Each tumor cell line was injected into 8 mice, and animals were maintained in a sterile animal facility. After 6 weeks, the mice were killed, and the lungs and liver were excised and examined for metastases. The experiments were repeated three times.

Finally, the presence of EIF5A2 was determined by an immunochemical analysis of 5-mm slices of formalin fixed paraffin-embedded tumor xenografts taken from nude mice.

### Statistical analysis

A Kaplan-Meier survival analysis was performed to assess the OS of the patients. A nonparametric Mann-Whitney test was used to analyze the relationship between miRNA expression levels and the various clinicopathologic characteristics. Differences between the groups were determined by a Student’s t-test (two tailed), and the relationship between EIF5A2 and miR-203 expression was explored by a Spearman’s correlation. All of the statistical analyses were performed with GraphPad Prism software (GraphPad Software Inc., La Jolla, CA, USA), and a P-value less than 0.05 was considered to be statistically significant.

## Additional Information

**How to cite this article**: Deng, B. *et al*. MiRNA-203 suppresses cell proliferation, migration and invasion in colorectal cancer via targeting of EIF5A2. *Sci. Rep.* **6**, 28301; doi: 10.1038/srep28301 (2016).

## Supplementary Material

Supplementary Information

## Figures and Tables

**Table 1:** Relationship between the clinicopathological factors and miR-203 expression in primary CRC.

Variable	miR-203 expression		P-value
	High (n = 32)	Low (n = 40)	
Age (years)			0.4771
≤50	18	18	
>50	14	22	
Gender			0.3427
Male	15	24	
Female	17	16	
Tumor size			0.0948
≤5 cm	22	19	
>5 cm	10	21	
Histological grading			0.3504
Well, moderate	19	19	
Poor	13	21	
TNM stage			0.0047
Stage I/II	23	15	
Stage III/IV	9	25	
Location			0.2341
Colon	17	27	
Rectum	15	13	
Lymph node status			0.0040
No metastasis	19	10	
Metastasis	13	21	

Well: well differentiated, moderate: moderately differentiated, poor: poorly differentiated, TNM: tumor-node-metastasis.

**Table 2:** Univariate and multivariate analyses of the association of prognosis with clinicopathologic parameters and miR-203 expression in CRC patients.

Variable	Univariable analysis		Multivariable analysis			
	**HR (95% CI**)	**P-value**	**HR (95% CI)**	**P-value**		
Age (<50/≥50)	0.741 (0.368–1.275)	0.362	–	–
Gender (male/female)	1.308 (0.694–2.147)	0.618	–	–
Differentiation (well/moderately, poorly)	1.324 (0.780–2.409)	0.356	–	–
Tumor size (<5 cm/≥5 cm)	1.182 (0.836–1.582)	0.367	–	–
Tumor location (Colon/Rectum)	0.946 (0.703–1.422)	0.306	–	–
Lymph node metastasis (present/absent)	6.545 (3.184–11.608)	<0.001	4.234 (3.104–8.332)	0.03
TNM stage (AJCC) (I-II/III-IV)	5.501 (2.243–13.462)	<0.001	5.043 (2.417–9.455)	0.007
miR-203 expression (high/low)	2.687 (1.204–5.764)	0.003	1.725 (1.139–3.053)	0.016

Bold values indicate statistical significance, P < 0.05, CI, confidence interval; HR, hazard ratio.
