# Supplementary material for: MiRNA-203 suppresses cell proliferation, migration and invasion in colorectal cancer via targeting of EIF5A2
Source: Sci Rep. 2016 Jul 4;6:28301. doi: 10.1038/srep28301 (PMC4931903; doi:10.1038/srep28301)
Supplement: Supplementary Information [file srep28301-s1.pdf]

MiRNA-203 suppresses cell proliferation, migration and invasion in colorectal cancer via targeting of EIF5A2

Biao Deng1*, Bin Wang1*, Jiaqing Fang2*, Xuchao Zhu3, Zhongwei Cao4, Qi Lin5, Lisheng Zhou1†, Xing Sun1†

Supplementary figures

Figure 1

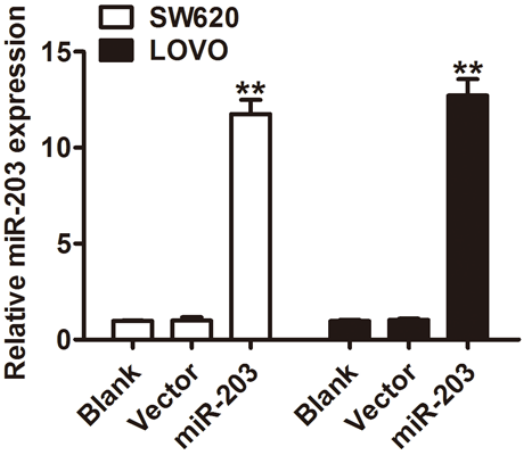

Figure 2

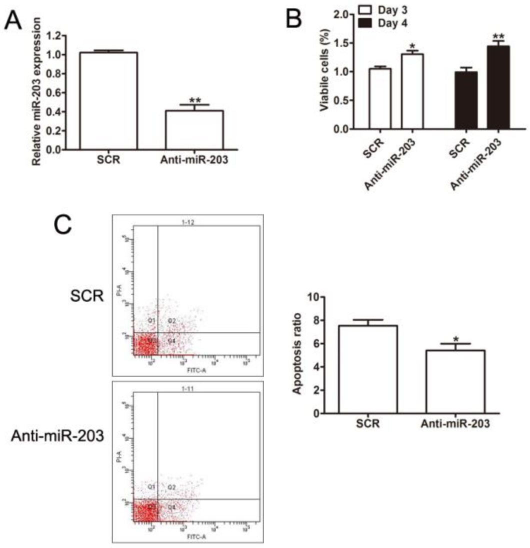

Figure 3

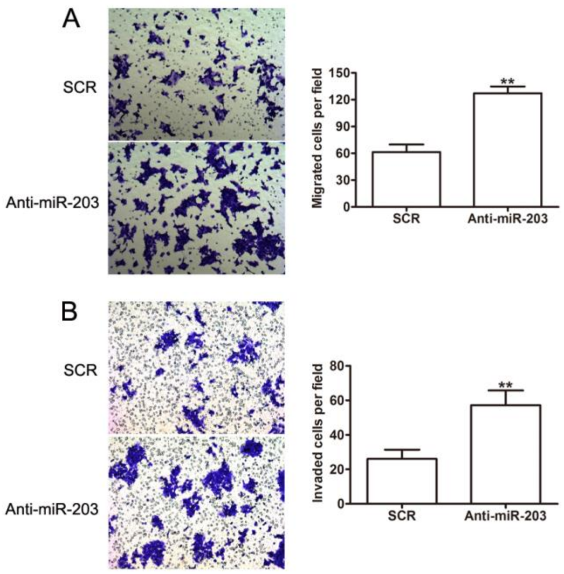

Supplementary figure legends

Supplementary Figure 1: Relative miR-203 levels were determined in SW480 cells by qRT-PCR 48 hours after transfection.

Supplementary Figure 2: MiR-203 inhibition enhances CRC cell proliferation and inhibits apoptosis. (A) Relative miR-203 expression levels in the SW480 cells transfected with the miR-203 inhibitor as determined by qRT-PCR. (B) MTT analysis of the cell growth rates of the SW480 lines transfected with the miR-203 inhibitor (Anti-miR-203) or the scramble control (SCR) after seeding. (C) Flow cytometry analysis of cell apoptosis in the SW480 cells.

Supplementary Figure 3: Silencing of miR-203 promotes cell migration and invasion in SW480 cells. (A) Representative micrographs (left) and quantification (right) of the migrated cells as determined by the migration assay in vitro. (B) Representative images (left) and quantification (right) of the invasive cells as determined by the matrigel invasion assay in vitro.
